# Root system ideotypes: what is the potential for breeding drought-tolerant grapevine rootstocks?

**DOI:** 10.1093/jxb/eraf006

**Published:** 2025-01-09

**Authors:** Sara Bernardo, Elisa Marguerit, Nathalie Ollat, Gregory A Gambetta, Clément Saint Cast, Marina de Miguel

**Affiliations:** EGFV, Univ. Bordeaux, Bordeaux Sciences Agro, INRAE, ISVV, 33882 Villenave d’Ornon, France; EGFV, Univ. Bordeaux, Bordeaux Sciences Agro, INRAE, ISVV, 33882 Villenave d’Ornon, France; EGFV, Univ. Bordeaux, Bordeaux Sciences Agro, INRAE, ISVV, 33882 Villenave d’Ornon, France; EGFV, Univ. Bordeaux, Bordeaux Sciences Agro, INRAE, ISVV, 33882 Villenave d’Ornon, France; EGFV, Univ. Bordeaux, Bordeaux Sciences Agro, INRAE, ISVV, 33882 Villenave d’Ornon, France; EGFV, Univ. Bordeaux, Bordeaux Sciences Agro, INRAE, ISVV, 33882 Villenave d’Ornon, France; Hong Kong Baptist University

**Keywords:** Agriculture, breeding, climate change, grapevine, ideotype, perennial crop, root system architecture, viticulture, water deficit

## Abstract

Adaptation to drought is one of the most important challenges for agriculture. The root system, along with its integration with the soil, is fundamental in conferring drought tolerance. At the same time, it is extremely challenging to study. The result is that investigations aimed at increasing crop drought tolerance have mainly focused on above-ground traits, especially for perennial species. In this review, we explore the root trait syndromes that would constitute drought-tolerant ideotypes, taking the example of grapevine as a model perennial grafted plant. We introduce and discuss the complexity of root trait interactions across different spatial and temporal scales considering their diversity, plasticity, and possible trade-offs. Finally, we review future approaches for discovering hidden root trait syndromes conferring drought tolerance, such as state-of-the-art root phenotyping technologies, the use of modeling as a tool to upscale root traits to the field, and new strategies to link genes to phenotypes. Together these integrated approaches can improve the breeding of drought-tolerant grapevine rootstocks.

## Teaser

Current knowledge of root trait syndromes contributing to drought tolerance provides essential information to conceive grapevine rootstock ideotypes for breeding programs.

## Introduction

Climate change is an enormous threat to agricultural systems worldwide. Among all of the challenges related to changes in climate, drought is clearly one of the most threatening. There is high confidence that the frequency and intensity of drought will increase in many areas of the world (IPCC, 2023). Drought is so menacing because it decreases crop productivity and increases mortality. As a result, there is a massive effort to develop drought-tolerant crops. The approaches are numerous, but a common foundation for successfully developing such crops is an understanding of the traits, and trait syndromes, that confer drought tolerance (Vadez *et al.*, 2024). The majority of the work to date has been focused on above-ground traits because the study of these traits is, practically speaking, much more feasible. At the same time scientists have been conscious of the fact that the root system (and its integration with the soil) is as, or perhaps even more, important in conferring drought tolerance.

**Fig. 1.**
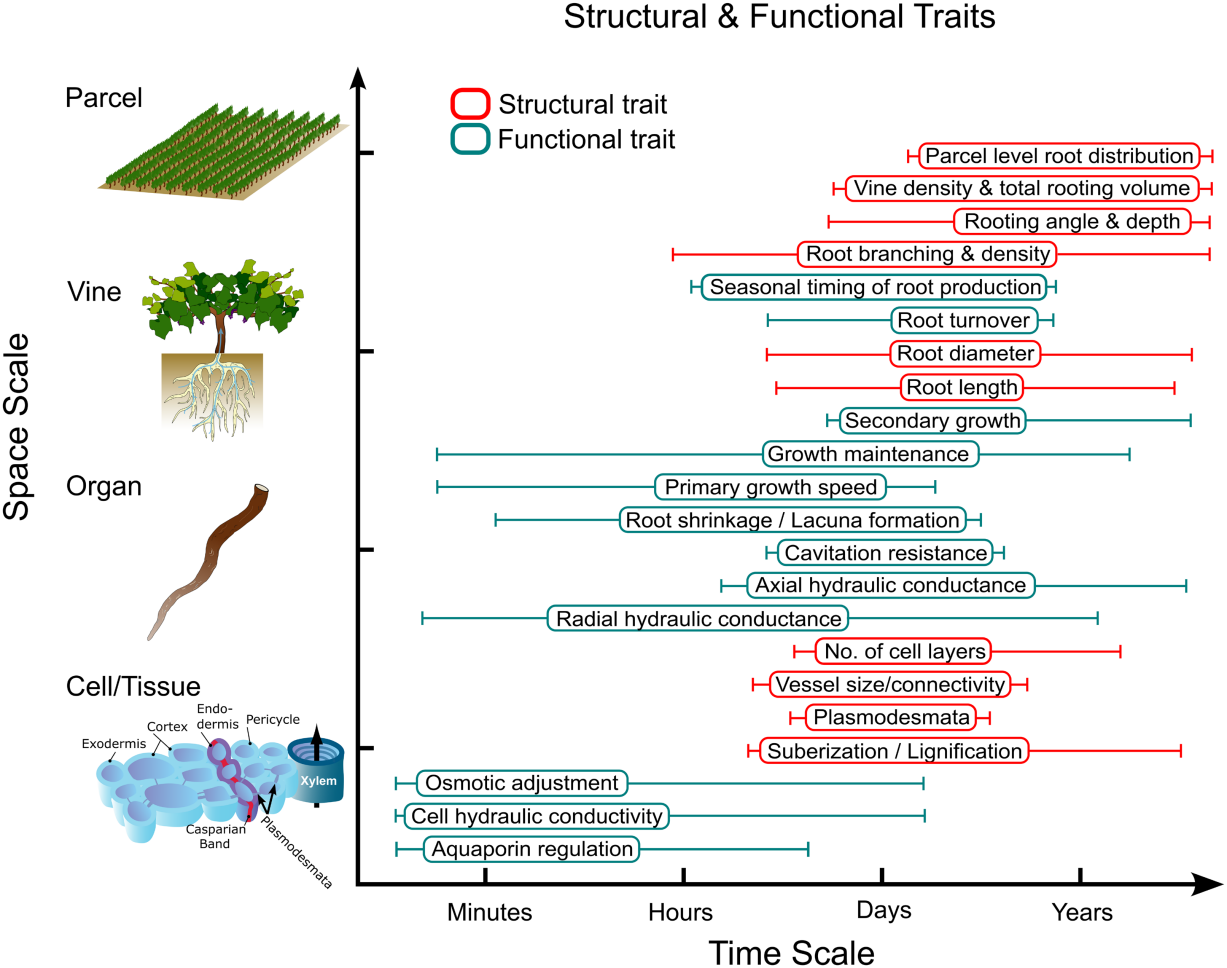
Root structural and functional traits that interact to bring about root system behavior under drought. One reason root system behavior is so challenging to study and predict is that it results from a complex interaction of structural (red) and functional (cyan) traits that interact across different scales of time (*x*-axis) and space (*y*-axis). Even single traits can manifest across different scales of time (whiskers). For example, a complex functional trait like radial hydraulic conductance can change over very short time frames (i.e. minutes to hours) via changes in aquaporin regulation and cell hydraulic conductivity, and over very long time frames (i.e. days to years) via changes in suberization, cell number, and the extent of secondary growth.

It is not just that roots are hard to access that makes their study more challenging. Root water uptake is influenced by a multi-scale combination of structural and hydraulic properties (Fig. 1). At the root system scale, root system architecture (RSA) defines the potential uptake sites within the soil (Maurel and Nacry, 2020). Hydraulic properties of individual roots (e.g. radial and axial conductivities) further constrain these uptake sites, defining a global hydraulic architecture of the plant. Changes in these hydraulic properties are thought to be an important target in breeding programs for drought-resistant crops (Rogers and Benfey, 2015). At a finer scale on the organ level, radial and axial conductivities can also be defined by structural and hydraulic properties. And on the cell/tissue scale, the radial conductivity of a root segment is influenced by the expression and localization of water channels (i.e. aquaporins), the formation of hydrophobic barriers, and/or the conductivity of plasmodesmata. Structurally, the radial conductivity is thought to be influenced by anatomical features such as the number of cell layers in the cortex, the size of cortical cells, the presence of aerenchyma, or the number and position of xylem vessels. Generally speaking, hydraulic properties are usually assumed to be controlled by the plant over the short or medium term, while structural features are assumed to be long-term. The integration and functioning of these different properties across scales controls root system water uptake and its cornerstone role in tolerating drought. It is this complexity, along with the physical inaccessibility of root systems, that makes their study so challenging.

In this review, we focus on grapevine as a model perennial crop and explore the potential role of root traits in breeding more drought-tolerant cultivars. We pose a question to which we do not currently know the answer: what root trait syndromes would maximize drought tolerance? This question guides a discussion of the complex trait interactions outlined above, their diversity and plasticity, and the potential conflicts and trade-offs across scales. Finally, we outline promising current and future approaches in phenotyping, modeling, and breeding that can help answer that question and lead to the development of new, more drought-tolerant cultivars.

## Perennial root system ideotypes

### Imagining the perfect perennial root system

So, what root trait syndromes (i.e. ideotypes) would maximize drought tolerance? This is a hugely challenging question, full of dichotomies and trade-offs, to which there is no single correct answer. The availability and distribution of natural resources profoundly shapes the morphology and physiology of plants. Plants adapt their development to maximize resource acquisition thus ensuring their survival and reproduction through time. Unlike annuals, which culminate their life cycle with senescence and death every season, perennials require a sequential cycling between vegetative and reproductive development each season. To assure their survival across many seasons, perennial root systems must adapt their morphology and physiology to maximize resource acquisition (Friedman, 2020).

It is almost certain that there is no single best ideotype. Here we will primarily focus on agrosystems, and we may expect perennial root system ideotypes to differ significantly in this context compared with natural ecosystems. In ecosystems, perennial root systems are generally deep (in comparison with annuals) and long-lived, with abundant woody roots that ensure the acquisition and storage of carbon, nutrients, and water to ensure growth and survival in a competitive environment year after year (Dawson *et al.*, 2024). During the domestication process of some perennial crops, the root system appears to have transformed towards a more resource-acquisitive behavior showing increased root branching, root length, and high nitrogen uptake (Pastor-Pastor *et al.*, 2019). This shift towards maximizing resource acquisition in domesticated perennial root systems can potentially create a trade-off, decreasing the storage capacity of the root system, and thus decreasing across-season resilience when compared with their wild predecessors. This transition was likely reinforced by higher input management systems that included irrigation and fertilization. In these high-input systems, reduced root development might even be advantageous, including phenotypes with fewer primary roots, a lower density of lateral roots, reduced growth sensitivity to resource availability, and a greater capacity to lose roots that do not contribute to water capture (Lynch, 2018). One example of this in maize are the ‘steep, cheap, and deep’ (SCD) ideotypes (Klein *et al.*, 2020). These ideotypes combines various architectural, morphological, and physiological features that improve water uptake and nitrogen while reducing the metabolic costs (Schmidt and Gaudin, 2017).

When searching for root system ideotypes of perennial crops, the question is how to ensure stable productivity and yields while at the same time ensuring survival under extreme environmental conditions (Vico *et al.*, 2016). To achieve this, the trade-offs between different functional strategies (e.g. explorative, conservative) need to be identified and well-understood (see also ‘Trait plasticity and its trade-offs’ below). To date, a large body of literature has concentrated on drought-tolerant root traits that are focused on increasing soil water availability. These traits include higher root hydraulic conductivity, delayed suberization, higher capacity to adjust root diameter, greater root area, deeper rooting, and higher osmotic adjustment capacity (Rowland *et al.*, 2023; Bonarota *et al.*, 2024). Additionally, steeper rooting angles to explore greater soil depths would help access water and mobile nutrients that quickly move through the soil profile. However, these traits may not be as advantageous in other contexts such as water limited shallow soils, and there may be additional limitations to rooting depth such as hypoxia, temperature, and soil toxicity (Lynch and Wojciechowski, 2015). Other authors have suggested that root system ideotypes should be able to balance the hydraulic redistribution along the soil profile, avoiding root loss in dry soils (Burgess *et al.*, 1998; Domec *et al.*, 2004; Bleby *et al.*, 2010). This can be achieved by limiting osmotic adjustment in drier soil patches (i.e. having a less negative turgor loss point), which would maintain greater turgor and growth in deeper roots (i.e. in wetter soil patches) while potentially favoring hydraulic redistribution to drier patches overnight (Bartlett *et al.*, 2022).

Identifying the best perennial root system ideotypes for specific agronomic contexts will remain an enormous challenge. It will require a deeper understanding of how trait syndromes function across different scales of time and space to maximize drought tolerance.

### Across different scales of time and space

The growing cycle of perennials requires integrating root growth over different time scales (i.e. within and across seasons) and space (i.e. throughout the soil volume), depending on environmental conditions (Fig. 1). Even under the challenging conditions of summer drought, plants manage to distribute their roots at different soil depths and radial distances from the plant. Season after season, these patterns of individual root growth result in a complex root system embodying roots of many different ages and orders with a variety of functions.

The root systems of perennial plants start with a fairly simple RSA, composed of a primary root (i.e. established from seed; order 1) or several adventitious roots (i.e. established from cuttings; order 1), which branch off into second-order roots (i.e. order 2) initiating third-order roots (i.e. order 3), and so on until some maximum number of branching orders (e.g. 10 branching orders were observed in maritime pine; Saint Cast *et al.*, 2019). This iterative branching takes place season after season, eventually resulting in a highly complex root system composed of thousands of roots of different types and orders, established over decades of growth. The different types and organization of roots observed in perennial species results in a diversity of morphological (e.g. short versus long, thin versus coarse, vertical versus horizontal) and functional (e.g. radial versus axial water transport) root properties. At the same time, segments of each root differ in anatomy (e.g. number of xylem vessels), morphology (e.g. diameter), and/or physiology (e.g. aquaporin activity) depending on age (Wells and Eissenstat, 2002; Vetterlein and Doussan, 2016; Heymans *et al.*, 2021). For example, gradients in axial and radial conductivity have been observed along the root length, corresponding to the increasing formation of apoplastic barriers (Enstone *et al.*, 2002), maturation of the xylem vessels (Steudle and Peterson, 1998), secondary growth (Gambetta *et al.*, 2013), and the decreasing expression and activity of aquaporins with age (Gambetta *et al.*, 2017).

Perennial crops establish their roots systems in a soil (both vertically and laterally) over many seasons, a process which takes ~7 years, on average, for grapevines (Champagnol, 1984). Following this establishment period the root system then invests in seasonal responses such as fine root turnover to maximize growth, yield, and plant survival (Munné-Bosch, 2014). The lifespan of fine roots is variable between different species and heterogeneous among individual fine roots, ranging in fruit crops from 60 d in apple to 300 d in citrus (Wells and Eissenstat, 2002). In adult grapevines, fine roots exhibit a wide range of lifespans, generally from a few weeks to several months. Fine root lifespan is related to the distribution of those fine roots within the soil profile and can be greatly influenced by soil management practices (e.g. under-trellis cover crops) (Centinari *et al.*, 2015). Fine roots occupying upper soil layers appear to have shorter lifespans, presumably due to varying soil moisture conditions (Wells and Eissenstat, 2002), while fine roots growing in deeper soil layers have longer lifespans (Anderson *et al.*, 2003).

Individual root growth also fluctuates over the season and depends greatly on climate conditions. In grapevine, growth occurs primarily in spring (between flowering and veraison) in Mediterranean-like and temperate climates, and in sub-tropical climates there is evidence that it primarily occurs post-harvest without a spring peak (Comas *et al.*, 2005, 2010). Regardless, the timing and speed of this process can be influenced by several factors, such as water and nitrogen availability, soil and canopy management practices, and/or mycorrhizal colonization. The effect of different rootstocks on the conferred vigor of root systems appears to involve shifts in the seasonal timing of growth. Bauerle *et al.* (2008) found that the high vigor 1103P and lower vigor 101-14 Mgt grapevine rootstocks displayed very different strategies regarding their seasonal patterns of root growth. The lower vigor rootstock 101-14 Mgt produced more roots during winter and displayed a slower accumulation of root density under drought, while 1103P produced more roots during summer and exhibited increased plasticity, allowing the exploration of wet soil patches. The authors suggest that these different seasonal growth patterns likely represent different drought adaptation strategies (without concluding which is more advantageous). Lower vigor rootstocks sustain root system development across the wet season (i.e. winter) maximizing access to soil water to better cope with summer drought, while high vigor rootstocks maintain growth and water foraging during the more drought-prone summer months.

There is natural interplant competition for water and other resources between individual roots, which occurs more frequently in crops that are planted at high densities (Schenk, 2006). Under limiting conditions, plants that exhibit higher specific root surface area and specific root length may be more competitive because they minimize the metabolic costs to fully explore a given soil volume (Hajek *et al.*, 2014). In this scenario, studies on forest ecosystems suggested that fine root trait plasticity might be pivotal in coping with below-ground competition (Fujii and Kasuya, 2008). Interplant root competition can occur through both exploitative competition (i.e. reduction of water and nutrients in a shared soil volume) and interference competition (i.e. blocking plant access to soil resources) (Schenk, 2006). Hence, adaptive and morphological responses at the fine root level might play an important role in reducing the exploitative competition in dry soil patches. Increased vertical root distribution with less branching can also improve plants’ ability to tolerate direct competition. One example examining communities of different species within a temperate grassland showed that while root systems with extensive lateral root development displayed strong exploitative competition, deep and sparsely branched root systems avoided this competition (Semchenko *et al.*, 2018).

As plants age, the root system tends to shift towards a more conservative-like behavior in perennial crops, investing in a variety of different root types at various depths to fulfill a variety of functions (Zhu *et al.*, 2023). Thus, in mature perennial plants, the root system is made up of a broad range of root ages, and the distribution of these different aged roots is an important feature in optimizing function while managing metabolic costs (Wells and Eissenstat, 2002; Comas *et al.*, 2010). Despite this diversity of root ages, roots are often grossly delimited into just two categories, young fine roots with high uptake capacity and less permeable coarse woody roots (i.e. roots that have undergone secondary growth and have a developed periderm or ‘bark’). Woody roots are thought to be less permeable to water and nutrients and typically one can assume that the larger the diameter the higher the xylem vessel area and axial water transport capacity (Vetterlein and Doussan, 2016). Fine roots are considered more permeable for water and nutrient uptake, but display a heterogeneous pattern for water uptake along their length, with more mature suberized root zones exhibiting lower uptake capacity (Gambetta *et al.*, 2013; Vetterlein and Doussan, 2016).

Generally speaking, fine roots are much more frequently studied when compared with woody roots. This is probably due to their importance for water and nutrient uptake. Because woody roots have a developed periderm (and decreased mycorrhizal associations), fine roots display greater water uptake rates (i.e. conductivity) per unit of root surface (Gambetta *et al.*, 2013). Nevertheless older woody roots still contribute to water uptake in many perennial systems including grapevine (Wells and Eissenstat, 2002; Gambetta *et al.*, 2013; Cuneo *et al.*, 2018). Determining the relative contributions of each different type of root to whole plant water uptake is the subject of speculation. The relative contribution is a function of both root portion specific hydraulic conductivity, that root portion’s surface area, and the surrounding soil hydraulic conductivity. Thus, given this complexity it is plausible that woody roots could make a large contribution to water uptake when their surface makes up a large proportion of the root system surface area (Cuneo *et al.*, 2018; Erktan *et al.*, 2018).

In summary, the contribution to water uptake and drought tolerance varies between root types (Protto *et al.*, 2024), orders, and/or segments in space and time. Fine roots can undergo higher root orders and may show potential secondary growth even in shallow soil layers, representing an advantage if the root system becomes damaged or fails to develop (Vetterlein and Doussan, 2016). In this context, we still need a better understanding of when (e.g. daily cycles, seasonal cycles, annual cycles), where (e.g. basal or apical root parts), how (e.g. regulation of hydraulic conductivity, osmotic adjustment, water redistribution), and which (e.g. root types or orders) roots contribute to water uptake and drought tolerance during the development of perennial plants. Understanding and integrating these factors will allow us to identify and characterize key root traits that could be instrumental in adapting to drought.

## Trait diversity and plasticity

### The spectrum of grapevine rootstock diversity

The grape industry relies on just a few well-characterized rootstock genotypes, and this limited diversity is primarily for historical reasons (de Andrés *et al.*, 2007). Today, 10 rootstock cultivars are used for ~90% of grafted grapevines across the largest grape-producing regions (Marín *et al.*, 2021). This low level of genetic diversity can seriously compromise plant adaptation, and consequently, challenge viticulture under uncertain future climate scenarios (Swarup *et al.*, 2021). The benefits of increased diversity for crop resilience have been well-documented for biotic resistance (i.e. avoiding resistance breakdown; Niks *et al.*, 2015), but diversifying the genetic mechanisms that confer adaptation to abiotic stresses such as drought could also be beneficial. Grapevine differs from many other domesticated perennial crops in that rootstock species are nearly undomesticated. They are either wild accessions or hybrids of just a few generations. There are a considerable number of rootstock genotypes conserved in germplasm banks; however, screening with molecular markers has revealed low levels of genetic diversity among them (de Andrés *et al.*, 2007). For example, all rootstocks are mainly hybrids or accessions from three North American *Vitis* species and the contribution from each species is often a single accession (*V. riparia* Gloire de Montpellier, *V. rupestris* du Lot, and *V. berlandieri* Resseguier II; Riaz *et al.*, 2019).

The low number of rootstock cultivars used worldwide contrasts with the reality that these rootstocks confer a large phenotypic diversity across many traits. These traits include those that constitute RSA and adaptation to different soil types, all of which have been studied for decades (Richards, 1983). The number and properties (e.g. tropism, ramification, or elongation) of root types and orders differ according to the plant genotype, propagation method (e.g. cutting *vs.* seedling), and environment (e.g. different soil water content) resulting in high phenotypic diversity in form, structure, and function between rootstock species, populations, or clones (Perry *et al.*, 1983; Fort *et al.*, 2017; Peiró *et al.*, 2020). For example, there is some evidence that the main roots of *V. riparia* form wide angles resulting in a shallow root system, while those of *V. rupestris* have narrow angles and can potentially penetrate deeper in the soil (Smart *et al.*, 2006). *Vitis berlandieri* is generally recalcitrant to the development of adventitious roots (rooting ability is slightly improved for grafted plants) but can develop a root system with drought-adapted functions such as a maintenance of water uptake and resumption of growth after re-watering (Barrios-Masias *et al.*, 2015; Cuneo *et al.*, 2021).

Apart from their ubiquitous use and high variation of expressed phenotypes, grapevine rootstocks have a narrow genetic foundation. Future research needs to address whether there is unexplored diversity able to improve root function under dry environments (Box 1). Several strategies can be adopted to move forward. On the one hand, we need to estimate the range of phenotypic diversity covered by lesser used genotypes in germplasm collections and newly bred rootstocks. It is crucial to evaluate the phenotypic variation for root traits (Fig. 1) in response to environmental stresses (i.e. concerning climate change scenarios) using novel phenotyping tools (e.g. omic data, from 2D to 4D root phenotyping technologies; Yıldırım *et al.*, 2018; Atkinson *et al.*, 2019). On the other hand, the exploration of wild relatives of grapevine rootstocks can introduce novel genetic diversity into breeding populations (Péros *et al.*, 2015). Wild accessions can harbor alleles favored by natural selection, a process that has tested many more allele combinations during species’ evolutionary history than humans will be capable of in a reasonable amount of time (Cortés and Barnaby, 2023). Consequently, genetic and functional characterization of wild *Vitis* spp. is a critical step for breeding next-generation rootstocks well-adapted to drought. Finally, we still lack knowledge on the genes controlling the development and function of perennial roots (Box 1). To address this question, genotype–phenotype association studies should be conducted on populations including both commonly used rootstocks and germplasm collections.

The genus *Vitis* (distributed in Asia, Eurasia, and North America) is a species complex with high levels of gene flow through frequent hybridization in nature (Morales-Cruz *et al.*, 2021). Nevertheless, it presents higher levels of species genetic variation than expected (Péros et al., 2011). This is also the case at the phenotypic level. For instance, high levels of phenotypic variation among species is frequently found for leaf and root morphological traits (Ickert-Bond *et al.*, 2018; Tandonnet *et al.*, 2018). Variation for hydraulic traits contributing to drought tolerance was similar among *Vitis* spp. than across *V. vinifera* cultivars (Dayer *et al.*, 2022). These results suggest that adaptation strategies to drought in cultivated grapevines may be as diverse as those we can find in nature, at least for above-ground organs. As rootstocks are poorly domesticated, we might expect the same for root drought adaptation strategies but, to our knowledge, this question has never been explored. In addition, high levels of phenotypic and genetic variation are found at the intra-specific level for *Vitis* spp., according to the different environments of their distribution and/or because of geographical reproductive barriers (Péros et al., 2021; Blois *et al.*, 2023). Therefore, it should be possible to introduce new *Vitis* spp. into breeding programs and to identify the best performing and/or robust accessions within species to breed rootstocks adapted to future climatic conditions (Aguirre‐Liguori *et al.*, 2022).

### Trait plasticity and its trade-offs

The diversity outlined above is hard-wired and innate, but equally important is the plasticity that root systems are capable of in order to acclimate to environment stresses such as drought (Fromm, 2019; Karlova *et al.*, 2021; Colombi *et al.*, 2024). Understanding plasticity could be of supreme importance for several reasons. First, any particular genotype will have some capacity for change, and thus its acclimation to a dynamic environment would be completely unpredictable without this knowledge. Second, there is no reason to believe a particular genotype with a particular trait syndrome would be optimal across every scenario. Different intensities, durations, frequencies, and cycling of drought episodes likely require different modes of acclimation, and the ability for a genotype to perform across a range of scenarios will be a direct result of its plasticity (Colombi *et al.*, 2024). Therefore, addressing environmental challenges using diversity without a consideration of plasticity could lead to unpredictable performance in the context of change.

Root systems acclimate to their environment across scales and have the ability to modify RSA and hydraulic properties (i.e. conductivity) in response to drought (Maurel and Nacry, 2020; Karlova *et al.*, 2021). This plasticity manifests within individual roots and then scales to the root system. Changes in branching patterns (leading to changes in RSA) are typically categorized into two phenomena: hydropatterning and xerobranching. Hydropatterning preferentially encourages lateral root formation into wet patches of soil (Bao *et al.*, 2014) while xerobranching suppresses lateral root formation in soil air pockets, thus promoting root elongation out of the dry soil patch (Orman-Ligeza *et al.*, 2018). Together with changing growth direction by xerotropism (an enhanced gravity response promoting downward curvature) and hydrotropism (promoting curvature towards wetter soil patches) root systems modify their RSA to maximize access to soil water under drought. Likewise in grapevine, changes in RSA appear to contribute to differences between rootstocks in maintaining gas exchange under drought (Peccoux *et al.*, 2018).

Hydraulic properties of grapevine roots and root systems are also very responsive to drought and these responses can be either non-reversible (i.e. plastic) or reversible (i.e. elastic) (Barrios-Masias *et al.*, 2015; Zhang *et al.*, 2016, 2020; Cuneo *et al.*, 2021). Plastic responses include structural changes such as changes in root anatomy (especially vascular), increased suberin deposition, and the formation of cortical lacunae that likely contribute to decreased hydraulic conductivity and hydraulic fusing (i.e. hydraulic disconnection of the root from the soil to protect against runaway dehydration; Cuneo *et al.*, 2016). Elastic responses include root osmotic adjustment (Bartlett *et al.*, 2022) and changes in hydraulic conductivity mediated by aquaporins (Gambetta *et al.*, 2012, 2017), which change remarkably quickly in response to drought and evaporative demand. For example, grapevine cultivars with different water use strategies appear to differentially regulate their root hydraulic conductivity, via aquaporin activity, during drought (Vandeleur *et al.*, 2009). The smaller diurnal reduction in root hydraulic conductance observed in the anisohydric-like cultivar Chardonnay under drought was linked with higher expression levels of specific aquaporin genes compared with Grenache, resulting in an increased contribution of the cell-to-cell pathway to the radial water transport (Vandeleur *et al.*, 2009). These plastic and elastic hydraulic responses can be synergistic in helping the root system acclimate to and recover from drought. In grapevine, cuttings from drought-tolerant and susceptible rootstocks were differentiated by structural and hydraulic changes; the drought-tolerant rootstock hydraulically disconnected from the soil faster under drought, but also re-established conductivity and growth faster after re-watering (Cuneo *et al.*, 2021).

Acclimation can be plastic or elastic, and myriad responses can lead to complex interactions that are not necessarily synergistic, resulting in potential trade-offs under different scenarios. These trade-offs can be broken down into two challenging questions that need to be addressed with future research. The first is whether more or less plasticity would be most beneficial with regard to a drought-tolerant root system (van der Bom *et al.*, 2020). Some argue for reduced plasticity so that the genotype preferentially develops a less branched, deeper root system that maximizes water (and nitrogen) uptake at depth (Lynch, 2018). However, this could produce a trade-off diminishing the root system ability to acquire less-mobile nutrients. In contrast, some amount of plasticity would be beneficial for adapting to within- and/or between-season heterogeneity in the soil profile. One example from Schneider and Lynch (2020) is short-duration plasticity that could enhance nutrient uptake in high input agricultural environments during the early season, but then reduced plasticity later in the season as discussed above—a ‘best of both worlds’ scenario. For long-lived, perennial crops like grapevine, some amount of plasticity is likely essential. The second question is what mix of plastic versus elastic responses would be the most efficient (Colombi *et al.*, 2024). This question is equally challenging in that differences in the number of stress cycles and energy costs of the acclimation interact to favor either a plastic or an elastic response. In summary, it is unlikely that any single genotype or particular trait syndrome will be best adapted to all scenarios. Therefore, breeding strategies will need to be targeted to specific environments and production contexts.

### Constraints and advantages of the grafted system

Understanding the genetic diversity and phenotypic plasticity in grafted plants is complex because the final phenotype depends on the interaction of two different genotypes. Much of the existing literature recognizes the impacts of rootstocks on scion vigor, leaf area, photosynthesis, and water and nutrient uptake (e.g. Paranychianakis *et al.*, 2004; Zhang *et al.*, 2016). These changes influence vine growth, yield, and fruit and wine quality. Although less studied, it is also true that the scion genotype can modify root development (Tandonnet *et al.*, 2010). However, the mechanisms through which rootstocks and scions interact are still unclear. Bidirectional exchanges of signaling molecules, such as hormones, metabolites, peptides, and nucleic acids are possible after grafting (Lu *et al.*, 2020). In grapevine, it has been shown that rootstocks can modify scion gene expression (Cookson and Ollat, 2013) and vice versa (Gautier *et al*., 2020) and that these changes are modulated by the environment (Harris *et al.*, 2023). In particular, drought-related modification of secondary metabolite pathways in berries is dependent on the rootstock genotype (Berdeja *et al.*, 2015), which likely impacts wine quality. Recently, bidirectional small RNA exchange has been suggested to contribute, potentially through epigenetic modifications, to the reciprocal gene expression changes between both graft partners (Rubio *et al.*, 2022). These exchanges are genotype-dependent and different graft combinations result in the activation of different molecular networks that tune drought-related miRNAs abundance and mode of action (Pagliarani *et al.*, 2017).

The most conspicuous limitation of grafting is the compatibility between genotypes. Creating successful grafts for specific combinations is a real limitation for grapevine nurseries (Loupit *et al.*, 2022; Tedesco *et al.*, 2022). Graft incompatibility goes beyond the initial graft formation and vascular integration, and also includes the long-term viability and productivity of the vine (Gautier *et al*., 2019; Loupit *et al.*, 2023). Numerous technical problems can reduce grafting success. Xylem reconnection between rootstock and scion after grafting is a key process conditioning the viability of the plant (Melnyk *et al.*, 2015; Marín *et al.*, 2022) and may be particularly important for water transport through the graft union. Although originally compatibility was thought to be largely dependent on species closeness, the body of scientific literature suggests that graft compatibility does not always match with phylogenetic relationships (Feng *et al.*, 2024). This is important because it means that the potential of grafting partners can be as broad as our ability to overcome the technical barriers during grafting. In this sense, grafting genotypes adapted to contrasting environments may allow the merging of different water and nutrient acquisition strategies in the same plant (see discussions above). However, the extent to which it is possible to break-down trade-offs between these strategies remains a matter of study (Bristiel *et al.*, 2019).

Taking into account these considerations, we could conclude that grafting complicates the understanding of grapevine drought responses. At the same time, it allows the combination of different water use strategies for the above- and below-ground organs. Therefore, understanding the mechanisms underlying the phenotypic variability resulting from rootstock by scion by environment interactions is essential for the development of drought-adapted rootstocks. Finally, grafting offers the possibility of biotechnological applications, such as transgrafting (i.e. the use of a genetically engineered rootstock to support a wild-type scion, or vice versa) (Albacete *et al.*, 2015). Transgrafting, could be applied as a strategy in grapevine to exploit new genes in the rootstock with potentially powerful effects on the scion.

## Futures perspectives

**Fig. 2.**
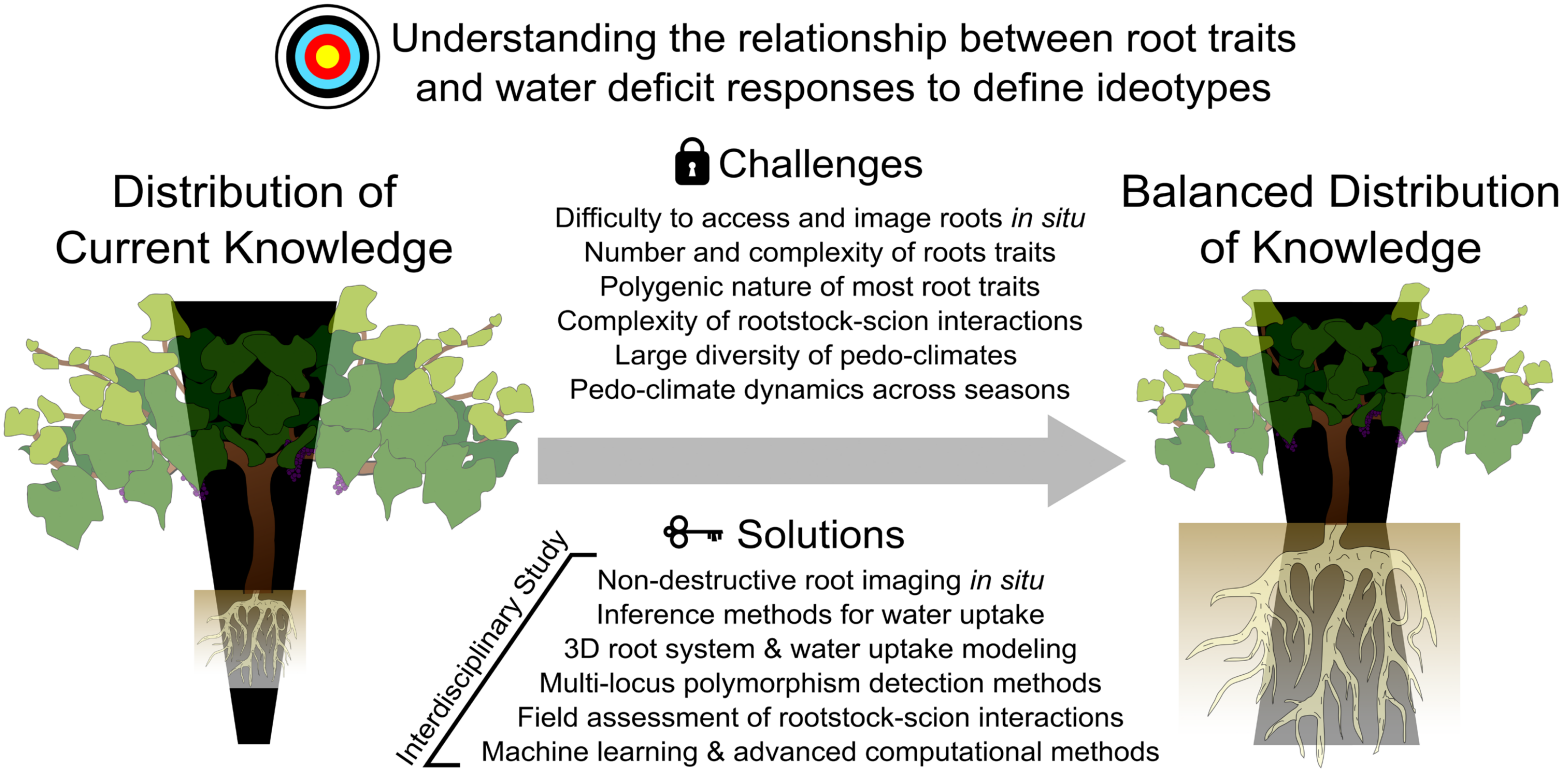
Challenges and solutions in defining drought-adapted grapevine root system ideotypes. Understanding the relationship between root traits and water deficit responses requires an interdisciplinary approach that balances knowledge on the above- and below-ground traits of the grafted system contributing to drought adaptation. The challenges of accessing and imaging roots in their natural environment, along with the number, complexity, and polygenic nature of root traits, as well as rootstock–scion interactions and climate variability, limit the study of root syndromes in the field. Combining interdisciplinary methodologies like 3D root system modeling, machine learning, and advanced computation, along with field assessment studies and multi-locus polymorphism detection, will likely help to address the current knowledge imbalance.

Root ideotype selection has been proposed as a means to develop and deploy rootstocks with improved traits to cope with more frequent and intense droughts and higher temperatures. However, designing these ideotypes poses numerous theoretical and practical challenges (Fig. 2): (i) drought tolerance results from multiple structural and functional traits at different spatio-temporal scales (Fig. 1); (ii) multiple different combinations of root traits may provide drought tolerance for each specific pedo-climatic condition; (iii) pedo-climatic conditions are as numerous as existing vineyards or parcelles, resulting in an unmanageable number of theoretical root system ideotypes; (iv) because perennials like grapevine are long-lived the pedo-climatic environment, and thus the rootstock ideotype, is likely to change significantly over the plant lifetime; and (v) it is currently extremely difficult to link specific genes to specific root traits (Box 1). In this context, we discuss future experimental and modeling approaches to identify key root traits, their physiological mechanisms, and underlying genetics to cope with drought in the future (Fig. 2).

### Revealing hidden trait syndromes through root phenotyping

The opaque nature of soils makes phenotyping roots and their plasticity *in situ* challenging. To overcome this problem, non-destructive and non-invasive techniques under controlled conditions with artificial growth media (i.e. hydroponics, aeroponics, gel plates, soil-filled tubes or pots; Atkinson *et al.*, 2019; Krzyzaniak *et al.*, 2021) have been used on young grapevines to examine how drought changes anatomical, morphological, and/or hydraulic properties of different grapevine rootstocks (Marguerit *et al.*, 2012; Gambetta *et al.*, 2013; Barrios-Masias *et al.*, 2015; Fort *et al.*, 2017; Cuneo *et al.*, 2021; Reingwirtz *et al.*, 2021; Bartlett *et al.*, 2022). Despite the advantages, studies under controlled conditions are extremely difficult to extrapolate to the complex and heterogeneous resource distribution experienced by mature grapevines in the field (e.g. presence of groundwater, bedrock, water table, or cemented horizon limiting root depth; Danjon *et al.*, 2005; Fan *et al.*, 2017; Grossiord *et al.*, 2017).

Root phenotyping under field conditions has seen significant advances in recent years. There has been significant development of sensor technologies to quantify root system development in a way that is non-destructive, repeatable, automatic, and autonomous (Wasson *et al.*, 2016; Nair *et al.*, 2023). One example are studies coupling soil moisture probes with minirhizotron systems. These transparent PVC tubes of ~2 m length are buried in the soil and function as an observatory with a camera system inside that scans the surrounding soil surface and roots. These imaging systems when paired with soil moisture probes allow the characterization of root length, branching, elongation, and/or mortality across different genotypes, soil types, and/or water regimes (Germon *et al.*, 2019; Svane *et al.*, 2019; Nair *et al.*, 2023). However, grapevine rootstocks can establish a deep root system in the absence of physical or chemical barriers (e.g. >6 m depth; Smart *et al.*, 2006), leading to experimental difficulties in phenotyping roots at depth. Alternative methods, referred to as inference methods, overcome this limitation by using indirect signatures of root distribution within the soil. For example, the isotopic composition of plant xylem water (δ^18^O and δ^2^H) can provide indirect evidence for the depth at which soil water is extracted and the absorptive root area distribution of the plant (Von Freyberg *et al.*, 2020). This technique was successfully used for different species and climates to quantify the distribution of water uptake depth among biomes and plant functional types (see the review of Bachofen *et al.*, 2024), and has also been used to understand seasonal changes in the depth of water uptake and the importance of groundwater for grapevines (Savi *et al.*, 2019). Coupling these phenotyping methods with drought experiments (e.g. precipitation exclusion; Asbjornsen *et al.*, 2018), different rootstock genotypes, and/or contrasting soil conditions (e.g. absence or presence of water table, different soil types) should help to unravel the drought-related traits and mechanisms involved in differing tolerance between grapevine rootstock genotypes.

### Using modeling to scale up root traits to the field

Given the complex interactions between root traits, water uptake, and the inherent development and plasticity of the root system over short and long periods of time, experiments will always have limitations. To cope with these difficulties, Draye *et al.* (2010) suggested developing *in silico* experiments to test how structural and functional root traits may increase drought tolerance under different pedo-climatic scenarios. In this context, models need to include an explicit 3D formulation of root system development in order to integrate available biological, physiological, and hydrological data across spatio-temporal scales. Some examples of these models are R-SWMS from Javaux *et al.* (2008) and MARSHAL from Meunier *et al.* (2020) which simulate water movement in the soil and roots simultaneously, considering the 3D root hydraulic architecture and heterogeneous water distribution. This allows one to quantify the contribution of each hydraulic, anatomical, and/or architectural root trait for thousands of phenotypes in different pedo-climatic conditions (Heymans *et al.*, 2021). However, to date, this type of modeling approach has only been developed and used for annual crops (e.g. maize), where high-throughput phenotyping platforms facilitate access to root traits to parameterize the models (Tardieu *et al.*, 2017; Atkinson *et al.*, 2019). Extending these types of models to perennial crops will need to take into account other processes such as carbon storage, water storage, water capacitance, and/or radial conductance of woody roots. Multidisciplinary approaches integrating phenotyping and modeling can fuel efforts to develop rootstocks with improved below-ground traits and increased drought tolerance (Fig. 2).

### Targeting specific genes

Genetic determinants have been found for most anatomical, morphological, and hydraulic root traits in annual crops (e.g. Courtois *et al.*, 2009; Ren *et al.*, 2022; Li *et al.*, 2024) but very few studies have addressed these same questions in perennial plants, and in grapevine specifically (Box 1). Multiple factors hamper genetic studies of root traits in perennial crops: long generation cycles, difficulty of self-pollination, high levels of heterozygosity, and the inaccessibility for phenotyping of below-ground organs with frequently destructive measures. As discussed above, the root traits and processes relevant for drought adaptation likely differ between annual and perennial crops, which hinders extrapolation of genetic results from annuals to perennials.

Studies have identified genes regulating RSA undergoing drought under controlled conditions, in perennials. There are several examples in poplar; *PdNF-YB21* positively regulates root growth and strengthens xylem lignification in roots, *PtabZIP1-like* enhances lateral root length and density (Dash *et al.*, 2017), and *PagWOX11/12a* promotes adventitious rooting and enhances root elongation (Wang *et al.*, 2020), all of them under drought stress. Additionally, transgenic apple plants overexpressing *MdMYB88* or *MdMYB124* had higher root vessel density and diameter, which improved hydraulic conductivity under long-term drought stress (Geng *et al.*, 2018). Transcriptomic studies on grafted grapevine rootstocks showed the regulation of sugar and protein transporters (SWEET and NRT1) correlated with drought-dependent RSA changes (Yıldırım *et al.*, 2018). Under water deficit, the up-regulation of two grapevine MYB41 orthologs was also linked to root suberin biosynthesis, export, and deposition putatively contributing to changes in root hydraulic conductivity (Zhang *et al.*, 2020). It is important to point out that none of these studies provides direct evidence that these genes can somehow confer drought tolerance in the field over the long lifespans of these perennial species.

Gene editing technologies hold promise for deciphering the mechanisms of root-specific genes conferring drought tolerance in perennial crops. However, the application of gene editing approaches for breeding drought-tolerant grapevine rootstocks is hampered by the complexity of root phenotypes and underlying genetic architecture involved in the drought responses. Complex root phenotypes are the result of polygenic architectures (Soriano and Alvaro, 2019; Chen *et al.*, 2021; LaRue *et al.*, 2022), which means that a high number of genes with small allele effects influence the final phenotypes. As a consequence, thousands of potential gene–gene interactions (both synergistic and antagonistic), modulated by heterogeneous environments, underlie the expression of root phenotypes under field conditions. In this context, addressing the desired changes in the whole plant behavior by modifying single genes is not feasible. For this reason, we believe the future of breeding for drought-adapted rootstocks will benefit from strategies that take into account these complex genome interactions.

### Unraveling beneficial allele combinations

Identifying quantitative trait loci and developing marker-assisted selection could be an efficient way to increase selection efficiency and boost the increase in trait performance obtained in root breeding programs (Wallace *et al.*, 2018). Classical genotype to phenotype association analyses are well suited for the detection of high-effect genes, but are not powerful enough to detect most causal variants for highly polygenic genetic architectures (Yang *et al.*, 2010). Multi-locus methods that consider a high number of genetic polymorphisms simultaneously have been recently developed (Segura *et al.*, 2012; Zeng *et al.*, 2018; Lloyd-Jones *et al.*, 2019). These methods improve our ability to detect low-effect variants and have the power to improve our understanding of complex root phenotypes. Coupling the knowledge of the genetic architecture of complex phenotypes with genome prediction approaches, such as genomic or phenomic selection, is an essential strategy for grapevine rootstock breeding programs. To our knowledge, these predictive approaches have not been tested for grapevine root traits, although promising results for above-ground traits have been recently obtained (Brault *et al.*, 2024). These predictive approaches can be further enhanced by interdisciplinary collaboration involving computational biology. Machine learning approaches have been used to help predict drought tolerance in grapevine rootstocks (Verslype *et al.*, 2023), and further advancements in modeling will certainly contribute to the identification and selection of drought-tolerant grapevine rootstock genotypes in breeding programs. Finally, future research on genotype–phenotype association in grapevine will undoubtedly involve the identification of genome structural variants, which may result in high effects on phenotypic variation, and the exploration of genetic diversity through pan-genome approaches (Cochetel *et al.*, 2023; Liu *et al.*, 2024).

### Rhizosphere microbial communities, soil abiotic characteristics, and root trait variation

Novel strategies aimed at improving soil resource acquisition are based on the selection of cultivars with specific root anatomy and RSA to favor the recruitment of beneficial edaphic microorganisms (Lynch, 2019). Natural variation in root phenotypes results in a diversity of niches for microbial associations in the rhizosphere, and at the same time, microbial traits influence specific root phenotypes (Galindo-Castañeda *et al.*, 2024). The synergies and trade-offs between roots and microbes are an emerging field and have rarely been studied in the field. Recent studies on perennial species, such as forest trees (Lasa *et al.*, 2022; Lin *et al.*, 2024) and grapevine (Darriaut *et al.*, 2022), demonstrated that intraspecific variation in plant roots significantly influences rhizosphere microbial communities, but we still need to link this differential recruitment to potential effects on plant performance (Lailheugue *et al.*, 2024), specifically under stressful conditions (Lasa *et al.*, 2022). Soil abiotic properties likely influence both the assembly of rhizosphere microbial communities and the expression of root phenotypes (Pérez-Izquierdo *et al.*, 2019; Lin *et al.*, 2024). For this reason, future research addressing the interactions between microbial communities and root traits should take soil properties into account.

## Conclusion

According to forecasts, drought will become a serious constraint for viticulture in the future (van Leeuwen *et al.*, 2024). Optimizing plant material will be an important strategy to adapt grapevines to more drought-prone viticulture regions. The development of drought-tolerant root systems will be fundamental to maintain productivity and sustainability. Achieving this goal is not trivial for perennial crops like grapevine, but the body of scientific work allows the identification of root strategies that can be targeted by breeding programs for root drought tolerance (Klein *et al.*, 2020). For example, some degree of phenotypic plasticity that allows the redistribution of roots within the soil profile in response to the edaphic environment seems to be a beneficial strategy to adapt to drought. As a grafted plant, grapevine offers the opportunity for breeding root trait syndromes to maximize drought tolerance independently from above-ground strategies aimed at optimizing fruit quality and yield. This will require a more complete understanding of the complex interactions between rootstock and scion in order to better model and predict whole plant drought response depending on the rootstock–scion combination. We argue that there is no single drought-tolerant root system ideotype, and furthermore that we cannot dissociate the breeding targets from the specific environment where they will ultimately be cultivated. Some open questions that future research should address include the role of phenotypic plasticity in root drought adaptation, the unexplored genetic diversity of grapevine rootstocks, the interaction of roots with edaphic abiotic and biotic factors (including the effects of managing practices), and an understanding of root responses to multiple, interacting environmental stressors. State-of-the-art root phenotyping and modeling approaches can contribute to answering these questions in the future.

Box 1. **Root-related quantitative genetics in grapevine**

It is a huge challenge to study the genetic architecture of root-related traits in grafted perennial crops. It requires the study of hundreds of individuals to identify correlations between genetic and phenotypic characteristics, which are complicated by interactions between the environment, the type of soil (or substrate if using potted plants), the innate heterogeneity of rooting, and the inaccessibility of the root system in the field. In grafted systems the rootstock genotypes are often neglected in ecophysiological and physiological studies, and almost never taken into account in quantitative genetic studies.

Only two studies have sought to characterize the root system architecture of grafted grapevine rootstocks (Tandonnet *et al.*, 2018; Blois *et al.*, 2023). The main characteristics of these studies are presented in Table 1. Moderate to high heritabilities are very promising characteristics for the study of these traits. However, few quantitative trait loci (QTL) were detected overall, highlighting an inability to identify all the genetic regions involved in the genetic determinism of root-related traits. Several candidate genes were included in the confidence interval of the QTL detected, although they still need to be validated using functional genomics approaches. Additionally, their allelic diversity could be studied across a larger breadth of grapevine rootstock diversity. Another quantitative genetic study carried out on own-rooted rootstock cuttings also allowed the identification of several QTL (Alahakoon and Fennell, 2023). This type of characterization provides important information for both breeding programs and grapevine nurseries, which ensures the production of rootstocks in the face of climate change.

**Table 1.** Main results issued from the two quantitative genetic studies carried out on grafted grapevine and root related traits

Study	Population	Scion	Number of genotypes/individuals	Type of soil		Traits measured	*H*²	Number of QTL	Percentage of variance explained per QTL
Tandonnet *et al.* (2018)	F_1_ pedigree population *V. vinifera* Cabernet-Sauvignon × *V. riparia* Gloire de Montpellier	Five scions (Cabernet-Sauvignon, Merlot, Petit-Verdot, Sauvignon blanc and Ugni blanc)	138/834	Gravelly sandy soil	Field conditions with high density	Aerial dry weight	0.44	1	11.5
						Root dry weight	0.61	3	10–10.4
						Root section	0.64	2	17.3–18.9
						Root number	0.7	1	20.7
						Root number per size category	0.52–0.65	4	12.1–20.4
						Aerial/root ratio	0.61	3	9.6–14.8
Blois *et al.* (2023)	*V. berlandieri* population for GWAS	Riesling	211/846	Medium soil	One-year potted vines without any limitation	Root dry weight	0.71	—	—
						Root number	0.82	1	0.4
						Root number per size category	0.56–0.79	6	0.6–8.5
						Sum of the diameter from all primary roots	0.73	—	—
						Average of the diameter from all primary roots	0.47	4	0.9–25.1

*H* ^2^, broad-sense heritability; QTL, quantitative trait loci.

In order to make significant progress in the future we need a better understanding of rootstock by scion by environment interactions. This knowledge will facilitate further study of the relationships between root-related traits and whole plant functioning, including drought tolerance. High-throughput phenotyping platforms have made it possible to phenotype large numbers of plants under controlled conditions (Tardieu *et al.*, 2018). For example, several loci of rootstock genomes controlling scion water use under drought have already been found in grapevine (Marguerit *et al.*, 2012). However, for perennial crops the characterization of potted plants for root-related traits is insufficient and field experiments are essential.

## Data Availability

No new data were generated in the production of this review.
